# Analysis of Blood Stem Cell Activity and Cystatin Gene Expression in a Mouse Model Presenting a Chromosomal Deletion Encompassing *Csta* and *Stfa2l1*

**DOI:** 10.1371/journal.pone.0007500

**Published:** 2009-10-19

**Authors:** Mélanie Bilodeau, Tara MacRae, Louis Gaboury, Jean-Philippe Laverdure, Marie-Pierre Hardy, Nadine Mayotte, Véronique Paradis, Sébastien Harton, Claude Perreault, Guy Sauvageau

**Affiliations:** 1 Institute for Research in Immunology and Cancer, University of Montreal, Montreal, Quebec, Canada; 2 Department of Medicine, University of Montreal, Montreal, Quebec, Canada; 3 Leukemia Cell Bank of Quebec and Division of Hematology, Maisonneuve-Rosemont Hospital, Montreal, Quebec, Canada; University Medical Center Groningen, Netherlands

## Abstract

The cystatin protein superfamily is characterized by the presence of conserved sequences that display cysteine protease inhibitory activity (e.g., towards cathepsins). Type 1 and 2 cystatins are encoded by 25 genes of which 23 are grouped in 2 clusters localized on mouse chromosomes 16 and 2. The expression and essential roles of most of these genes in mouse development and hematopoiesis remain poorly characterized. In this study, we describe a set of quantitative real-time PCR assays and a global expression profile of cystatin genes in normal mouse tissues. Benefiting from our collection of DelES embryonic stem cell clones harboring large chromosomal deletions (to be reported elsewhere), we selected a clone in which a 95-kb region of chromosome 16 is missing (*Del^16qB3Δ/+^*). In this particular clone, 2 cystatin genes, namely *Csta* and *Stfa2l1* are absent along with 2 other genes (*Fam162a*, *Ccdc58*) and associated intergenic regions. From this line, we established a new homozygous mutant mouse model (*Del^16qB3Δ/16qB3Δ^*) to assess the *in vivo* biological functions of the 2 deleted cystatins. *Stfa2l1* gene expression is high in wild-type fetal liver, bone marrow, and spleen, while *Csta* is ubiquitously expressed. Homozygous *Del^16qB3Δ/16qB3Δ^* animals are phenotypically normal, fertile, and not overtly susceptible to spontaneous or irradiation-induced tumor formation. The hematopoietic stem and progenitor cell activity in these mutant mice are also normal. Interestingly, quantitative real-time PCR expression profiling reveals a marked increase in the expression levels of *Stfa2l1/Csta* phylogenetically-related genes (*Stfa1*, *Stfa2*, and *Stfa3*) in *Del^16qB3Δ/16qB3Δ^* hematopoietic tissues, suggesting that these candidate genes might be contributing to compensatory mechanisms. Overall, this study presents an optimized approach to globally monitor cystatin gene expression as well as a new mouse model deficient in *Stfa2l1/Csta* genes, expanding the available tools to dissect cystatin roles under normal and pathological conditions.

## Introduction

Chromosomal deletions are a powerful tool to assess the biological functions and genetic interactions among contiguous genomic elements irrespective of whether they are protein-coding or non-coding. We have previously developed a retroviral-based Cre-*loxP* system to engineer chromosomal deletions in mouse embryonic stem cells (ESCs) that can successfully be submitted to *in vitro* and *in vivo* haploinsufficient screens [1]. Among the mutant ESC clones previously generated, the clone 7–30 is characterized by a 95-kb haploid deletion mapped on chromosome 16 (*Del^16qB3Δ/+^*) that does not perturb its potential to differentiate normally into embryoid bodies [1]. Mapping of the rearrangement breakpoints by inverse-PCR revealed that the deletion spans four genes: *Fam162a* (partially deleted), *Ccdc58* (completely deleted), *Csta* (completely deleted), and *Stfa2l1* (completely deleted) [1]. *Fam162a* codes for the mitochondrial protein Fam162a (also known as growth and transformation-dependent protein), thought to be a death-inducing effector downstream of hypoxia-inducible factor 1α [2]. *Ccdc58* codes for the Coiled-coil domain-containing protein 58 [3], which currently has no known functions. Microarray-based expression profiling suggests that *Fam162a* and *Ccdc58* are expressed during mouse embryogenesis and in a wide variety of tissues of adult animals [4]. Enhanced Fam162a cytoplasmic expression is detected in human gastric carcinoma and adenoma compared to normal mucosa but the role of this protein in tumorigenesis is undetermined [5]. The two other deleted genes, *Csta* (protein: Cystatin A, also known as Stefin A) and *Stfa2l1* (protein: Stefin A2-like 1), are part of a stefin gene cluster (n = 10 genes: *Csta*, *Stfa2l1*, *Gm5483*, *Gm5416*, *2010005H15Rik*, *Stfa1*, *BC117090*, *BC100530*, *Stfa2*, *Stfa3*) present on mouse chromosome 16 [3]. The stefin proteins, a subgroup (Type 1) of the cystatin superfamily, act as cytoplasmic inhibitors of cysteine proteases such as cathepsins [6], [7]. Given their inhibitory role toward cathepsins, stefin proteins could regulate antigen presentation processes involved in immune response and autoimmune diseases [7], [8]. Microarray-based expression profiling reveals that *Stfa2l1* is mainly expressed in early embryos (E6.5), in the umbilical cord, and in the bone, bone marrow, spleen, and thyroid of adult mice [4]. *Csta* expression is observed in the epidermis, bone marrow (subset of myeloid cells), spleen (cells dispersed in red pulp), and oesophagus of adult mice [9], [10]. *Csta* expression is modulated in a variety of human cancers [10], [11], [12], [13], [14]. The functional role of Cystatin A in these cancers has not been completely elucidated. In a human esophageal squamous cell carcinoma xenograft model or in a syngenic murine model of mammary gland tumorigenesis, tumor cells overexpressing *Csta* demonstrate a reduced capacity to form lung or bone metastasis respectively, suggesting that Cystatin A may act as a tumor suppressor within these contexts [15], [16]. So far, no mouse models deficient in *Fam162a*, *Ccdc58*, *Csta*, or *Stfa2l1* have been described.

In the present study, we have generated homozygous null *Del^16qB3Δ/16qB3Δ^* mice and have shown that *Csta* and *Stfa2l1* are dispensable for viability, fertility and hematopoietic stem cell activity. We also document the expression of most cystatin genes in normal hematopoietic tissues and provide evidence for compensation by phylogenetically-related family members in *Del^16qB3Δ/16qB3Δ^* cells.

## Materials and Methods

### Ethics statement

All animals were handled in strict accordance with good animal practice as defined by the relevant national and/or local animal welfare bodies, and all animal work was approved by the appropriate committee (University of Montreal animal ethics committee: Comité de Déontologie de l'Expérimentation sur les Animaux de l'Université de Montréal. Protocols 08-113 and 08-159). Animals were housed in ventilated cages and provided with sterilized food and acidified water under veterinary supervision.

### Maintenance and engineering of mouse ESCs

Chromosomal deletions were previously engineered in R1 ESCs [17] with a retroviral-based Cre-*lox*P system [1]. ESCs were maintained as described [1]. The chromosomal deletion found in the karyotypically stable ESC clone used for this study (clone 7–30) was previously mapped on mouse chromosome 16 (36071173–36166797, NCBI mouse Build 37) by inverse-PCR [1].

### Mutant mouse generation and genotyping

Chimeric mice were generated by injecting the engineered ESCs into C57BL/6J morulas and blastocysts. Two highly chimeric males were crossed with C57BL/6J females to achieve germline transmission of the mutant allele. Heterozygous mutant mice were intercrossed to establish a stable colony of homozygous mice. Genotyping was performed by PCR using genomic DNA extracted from tail clips (lysis with of 100 mM NaCl, 10 mM Tris pH 8.0, 25 mM EDTA pH 8.0, 0.5% SDS, and 2.5 ug/ml Proteinase K followed by phenol-chloroform extraction and ethanol precipitation) or Sigma REDExtract-N-Amp™ Tissue PCR kit (primers are described in the Table S1). Southern blot analysis was performed as previously described [18] with *Eco*RI-digested genomic DNA from tail clips (*Eco*RI cleaves once inside the recombined retroviral vectors) and a neomycin probe.

### PCR analysis of the chromosomal deletion

The deleted chromosomal segment was confirmed by PCR performed on genomic DNA extracted from mouse tail clips (see above) using a set of PCR assays described in Table S2.

### Mouse response to stimuli (visual, aural, pain) and neuromuscular function

The visual response was evaluated by a menace response test whereby an immediate reaction from the mouse is provoked by quickly moving a finger towards the mouse. The aural response was evaluated by observing that the mouse flicked its ears backwards in response to aural stimulus. The response to pain was evaluated by its response to tail pinch stimulus. Neuromuscular function was evaluated by the grip strength test where mice grasp a metal grid when lifted by the tail, as well as by the mouse's ability to support its hind end.

**Clinical hematology, serum biochemistry and urinalysis** based on standardized assays were performed by the McGill University Animal Resources Centre (http://www.medicine.mcgill.ca/arc/).

### Histology

E12.5, E14.5, E16.5 fetuses and dissected organs from adult mice (3 months of age) were fixed overnight in a 10% buffered formalin solution (Sigma-Aldrich) at room temperature, dehydrated, paraffin-embedded, and sectioned according to standard procedures. Histological sections stained with hematoxylin and eosin were examined by a veterinary pathologist (LG). Peripheral blood cytosmears and bone marrow cytospins (bone marrow cells flushed with a 21-gauge needle in 2% FBS in PBS) were stained with Wright's stain and examined by a hematologist (GS).

### Flow cytometry analysis of cells present in hematopoietic organs

Bone marrow cells were isolated as mentioned above. Spleen and thymus cells were gently dissociated through a nylon mesh in 2% FBS in PBS. Conjugated primary antibodies recognizing cell surface epitopes used in these studies are: for erythroid and megakaryocytic lineages: Ter119-APC (BioLegend, San Diego, CA), CD71-PE (eBioscience, San Diego, CA), CD41-FITC (BD Bioscience Pharmingen, Franklin Lakes, NJ); for myeloid lineages: Cd11b-PE (BD Bioscience Pharmingen, Franklin Lakes, NJ), Gr1-FITC (BD Bioscience Pharmingen, Franklin Lakes, NJ); for B cell lineages: B220-APC (eBioscience, San Diego, CA), CD43-FITC (BD Bioscience Pharmingen, Franklin Lakes, NJ); for T cells of the bone marrow and spleen: CD4-FITC (BD Bioscience Pharmingen, Franklin Lakes, NJ), CD8-APC (BD Bioscience Pharmingen, Franklin Lakes, NJ); for hematopietic stem cell-enriched populations of the bone marrow: cKit-PE-Cy7 (eBioscience, San Diego, CA), Sca1-PE (BD Bioscience Pharmingen, Franklin Lakes, NJ), CD150-PE (Bioloegend, San Diego, CA), CD48-FITC (BD Bioscience Pharmingen, Franklin Lakes, NJ), Ter119-APC, Gr1-APC, and B220-APC; for T cells and other cells in the thymus: cKit-PE-Cy7, CD25-APC, CD4-APC-Cy7, CD8-PerCP, TCRß-FITC, CD19-APC, CD45.2-FITC, CD11c-PE-Cy7, Ly51-APC, IAb-PE. The lineage negative population of the thymus was stained with a cocktail of biotinylated antibodies (TCRαβ, TCRγδ, CD11b, CD11c, NK1-1, CD8, B220, Ter119, GR-1 and CD3) which were then labeled with APC-Cy7-Streptavidin. All antibodies used in thymus experiments were from BD Pharmingen, Franklin Lakes, NJ. Stained samples were processed using an analytical flow cytometer (BD LSR II, BD Biosciences, San Jose, CA, USA) and data analyzed with FlowJo software (Tree Star Inc., Asland, OR, USA).

### *In vitro* clonogenic progenitor assays

These assays were performed as previously described [19] with cells isolated from the bone marrow and the spleen of two representative mice for each genotype (n = 2 wild-type, n = 2 heterozygous, and n = 2 homozygous mutant mice). Cells were seeded into semi-solid media (1 ml, in duplicate) at the following densities: 4×10^4^ bone marrow cells/ml and 5×10^5^ spleen cells/ml for myeloid clonogenic progenitor assays, and 2×10^5^ bone marrow cells/ml and 2×10^6^ spleen cells/ml for B-lymphoid clonogenic progenitor assay.

### Competitive bone marrow reconstitution assay

E14.5 fetal liver cells were isolated from two independent wild-type Pep3b mice (CD45.1^+^) and two independent homozygous mutant mice backcrossed with C57BL/6J mice for one generation (CD45.2^+^). In two independent settings, sublethally irradiated Pep3b mice (800 cGy, n = 4–5 mice) were transplanted with 1×10^5^ bone marrow helper cells (isolated from Pep3b mouse) along with the following mixture of fetal liver cells: 0.5×10^5^ wild-type + 2×10^5^ homozygous mutant. Peripheral blood was collected from the tail vein at 12 weeks post-transplantation, erythrocytes were lysed as described [20], and the percentage of CD45.1^+^ and CD45.2^+^ circulating cells was determined by flow cytometry (see above) using specific conjugated primary antibodies (CD45.1-APC and CD45.2-FITC, BD Pharmingen , Franklin Lakes, NJ USA, USA).

### Susceptibility to irradiation-induced tumors

Mutant mice were backcrossed once in a C57BL/6J background and heterozygous mutant mice were intercrossed. Cohorts of littermate animals (n = 13 wild-type, n = 30 heterozygous, n = 15 homozygous mutant mice) were sublethally irradiated (whole body irradiation, 600 cGy of ^137^Cs γ-irradiation) at 5 weeks of age and monitored for tumor formation.

### Cystatin phylogeny inference

Amino acid sequences of murine members of the cystatin protein family (including Type 1, Stefins; Type 2, Cystatins; but excluding Type 3, Kininogens) were collected using NCBI's Entrez Gene database [21]. The sequences were aligned with ClustalW2 [22] using the Gonnet250 substitution matrix. Adequate parameters for phylogeny inference were obtained by submitting the produced alignment to the ProtTest evolutionary model selection software [23]. Maximum Likelihood-based phylogeny was then inferred using PhyML [24] using the parameters suggested by ProtTest based on the Akaike Information Criterion framework (AIC) score. Namely: the WAG evolutionary model along with gamma correction and amino acid frequency correction. Finally, robustness of the obtained tree was evaluated by running 1000 bootstrap iterations.

### Quantitative real-time PCR (qRT-PCR)

Mouse tissues were disrupted in Trizol using a Polytron homogenizer and total cellular RNA was isolated according to the manufacturer's instructions (Invitrogen, Burlington, ON, Canada). Two micrograms of RNA was reverse transcribed using Superscript II Reverse Transcriptase (engineered version of M-MLV Reverse Transcriptase) and random hexamers, according to the manufacturer's guidelines (Invitrogen Burlington, ON, Canada). Gene expression was assessed by qRT-PCR using the Roche Light Cycler 480 with Roche Universal ProbeLibrary assays, SYBR green assays (Stefin genes) or TaqMan assays (endogenous control genes) as described in Table S3 and Text S1. Reactions were performed in duplicate in 384 well plates for 50 amplification cycles (95°C 10 s; 60°C 10 s; 72°C 10 s). Primers for SYBR green assays of stefin genes were carefully designed to include nucleotides specific to the desired gene as determined by multiple sequence alignment (Text S1). Specificity of SYBR green-based assays was confirmed by melting curve analysis and sequencing of amplicons. Reference gene assays (*Gapdh* and *ß-actin*) were purchased from ABI (20× primer-probe mix, VIC labeled). All other gene expression assays were designed using the Roche Universal ProbeLibrary assay design software (Advanced primer3 settings) and were tested for maximum efficiency by standard curve analysis (slope = 3.1–3.6). For a given cDNA sample, threshold cycle (Ct) values were determined using the Advanced Relative Quantification algorithm for each target gene (Ct_target_) as well as *Gapdh* and *ß-actin* endogenous reference genes (Ct_reference_). cDNA control samples present on each plate insured reliability of Ct values generated for each target and reference gene studied, as expected from consistent PCR reactions. Heatmaps were generated to represent the relative expression of each target gene normalized to endogenous reference genes (e.g. ΔCt = Ct_target_−average Ct_reference_) for each given cDNA sample (average from duplicate assays performed with cDNA derived from 2 independent adult mouse tissues, from a pool of embryos, or from 1–4 independent fetal livers, as indicated in the legends). Heatmaps of the qRT-PCR data were produced using the R ‘heatmap’ function. Hierarchical clustering of the expression profiles for both genes and samples was performed using the complete linkage method and euclidian distance metric. To compare relative gene expression in selected tissues of wild-type mouse (calibrators for normalization) and of mutant mouse (samples), reactions were performed in triplicate and the comparative Ct method was employed (2^−ΔΔCt^; ΔΔCt for a given gene in a given tissue = ΔCt_sample_−ΔCt_calibrator_). Relative gene expression values for calibrators were set to 1.

## Results

### Generation of a homozygous mutant mouse line and expression profiling of cystatin genes

**Figure 1:**
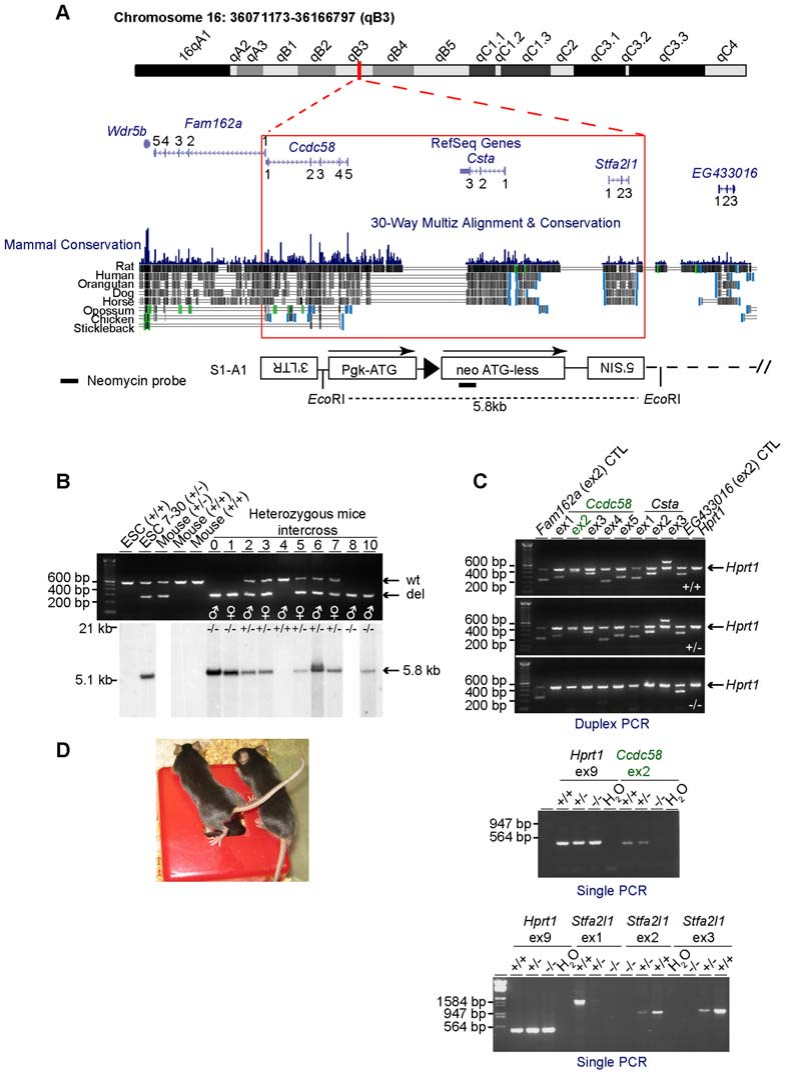
Generation of mutant mice. (A) Representation of the 95-kb haploid chromosomal deletion found in an ESC clone (7–30) engineered by a retroviral-based Cre-*loxP* system [1]. The schema was adapted from UCSC Genome Browser (http://genome.ucsc.edu/) [3]. Mammal conservation (blue histogram) represents a evolutionary conservation measure across 20 placental mammal genomes. In addition, 8 pairwise alignments of vertebrate genomes to the mouse genome can be found underneath (greyscale density plots) [3]. Also indicated, the recombined provirus originating from the recombination of two independent proviruses (A1 and S1, see reference [1] for details). The coupling of the *Pgk* (murine phosphoglycerate kinase promoter)-ATG in S1 to the neomycin (neo ATGless) gene in A1 allowed the selection of recombinant ESC clones. LTR, long terminal repeat; SIN, long terminal repeat containing a deletion in the U3 region; ▸, *LoxP* site. (B) PCR-mediated genotyping (top panel) performed with genomic DNA extracted from wild-type (+/+) ESCs, engineered ESCs (+/−), and mouse tail clips from littermate animals obtained by intercrossing heterozygous (+/−) mice. Southern blot analysis performed with a neomycin probe (bottom panel, also see Figure 1A for *Eco*RI enzymatic restriction digest pattern and neomycin probe localization) confirming the presence of the engineered allele in heterozygous or homozygous (−/−) mutant animals. wt, wild-type allele; del, deleted allele. (C) PCR products spanning exons (ex) or intron-exon boundaries confirmed the loss of the mapped genomic region in a representative homozygous animal (see Figure 1A for exon numbering and Table S2 for PCR assay details). CTL, control. PCR for the *Hprt1* gene was performed as a positive control in duplex or in parallel. (D) Representative photo of adult wild-type and homozygous *Del^16qB3Δ/16qB3Δ^* mice.

We took advantage of a previously engineered mouse ESC line containing a chromosomal deletion to assess the *in vivo* biological functions of *Csta* and *Stfa2l1* (stefins) as well as *Fam162a*, *Ccdc58*, and associated intergenic regions (Figure 1A). The 95-kb chromosomal deletion found in these ESCs specifically spans the evolutionarily conserved coding sequences of *Ccdc58*, *Csta*, *Stfa2l1* as well as the first coding exon of *Fam162a* (Figure 1A). These cells were used to generate chimeric mice that transmitted the chromosomal deletion successfully through the germline (not reported before). Heterozygous animals appeared phenotypically normal and could be intercrossed. At the time of weaning, littermate animals of all expected genotypes were confirmed by genotyping (Figure 1B) and were present at a Mendelian ratio (32% wild-type, 44% heterozygous, and 24% homozygous; n = 100 monitored animals). PCR assays confirmed the loss of genomic exons or adjacent regions throughout the area covered by the chromosomal deletion in homozygous animals (Figure 1C). All of these animals reached adulthood without phenotypic anomalies (Figure 1D and characterization below).

**Figure 2:**
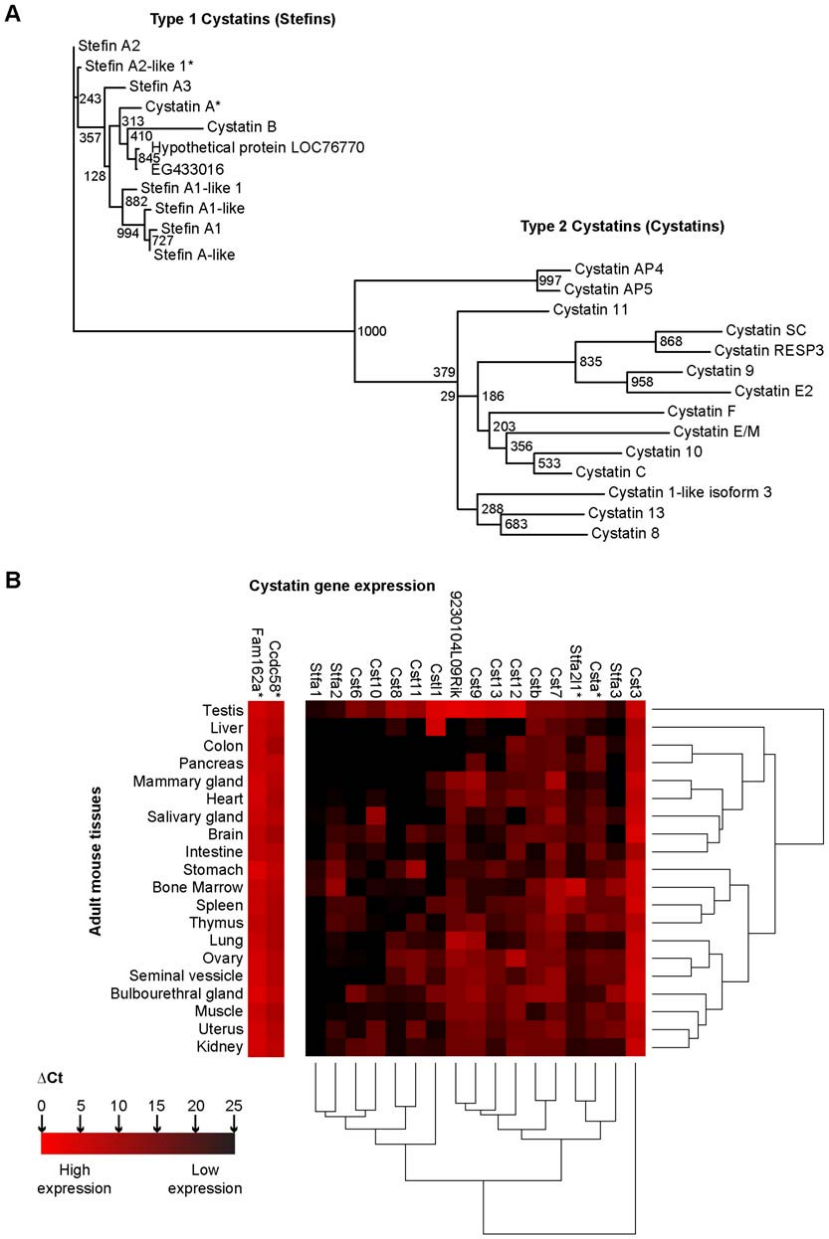
Comprehensive analysis of type 1 and 2 mouse Cystatins. (A) Phylogeny inference of type 1 and 2 cystatins based on amino acids sequence comparison. Numbers represent the degree of confidence of the nodes, as evaluated by the bootstrap test (highest degree of confidence: 1000). *Genes deleted in *Del^16qB3Δ/+^* ESCs. (B) ΔCt heatmap representing the expression profiles of *Fam162a*, *Ccdc58*, and several cystatin genes in the indicated tissues of adult mice (n = 2 independent tissues analyzed in duplicate, see Material and Methods section for details). Note that the qRT-PCR assay for *Stfa1* also detects *BC117090* and *BC100530* transcripts. ΔCt above 15 represents weak gene expression or no expression; the expression cut-off varies according to each specific qRT-PCR assay (between ΔCt 15–25).

In order to get an accurate perspective of the mouse stefin genes within the cystatin superfamily, a list of cystatins and cystatin-like candidates was extracted from NCBI's Entrez Gene database [21] (Table 1). Remarkably, genes encoding type 1 (e.g. stefins) and type 2 (e.g. cystatins) cystatins are grouped in two main clusters mapped on mouse chromosome 16 and 2, respectively (Table 1), with the exception of *Cstb* on chromosome 10 and *Cst6* on chromosome 19. Phylogeny inference based on amino acid sequences highlighted the high degree of sequence similarity across stefin variants, which appear to have evolved from Stefin A2 (Figure 2A). qRT-PCR assays were designed to establish an integrated transcriptional profile of *Csta* and *Stfa2l1* within the cystatin gene network and to monitor *Fam162a* and *Ccdc58* expression in normal mouse tissues (Table S3). Given the high degree of sequence similarity between some of the stefin members (Figure 2A), gene sequence alignments were required to design SYBR green-based qRT-PCR assays specific to *Stfa1* (detecting also *BC117090* and *BC100530*), *Stfa2*, *Stfa2l1*, and *Stfa3* (Text S1 and Table S3). To confirm the specificity of these assays, amplicons were sequenced to distinguish transcript unique nucleotides (highlighted nucleotides in Text S1). TaqMan-like Roche Universal ProbeLibrary assays were designed to monitor *Csta*, *Cstb*, type 2 cystatin genes, *Fam162a*, and *Ccdc58* (Table S3). Global expression analysis was performed with RNA extracted from developing mouse embryos and E14.5 fetal liver cells (Figure S1) as well as various tissues from male and female adult mice (Figure 2B). *Fam162a*, *Ccdc58*, and *Cst3* were highly expressed in all analyzed tissues (Figure S1 and 2B). Expression of several cystatin genes (type 1 and 2) was detected during embryogenesis (Figure S1). They were also expressed in various combinations in the tissues of adult animals, ranging from few to several expressed members depending on the tissue analyzed (for example, see cystatin gene expression in liver versus testis, Figure 2B). Within the stefin subgroup, *Csta*, *Cstb*, and *Stfa3* were expressed in a variety of tissues while *Stfa1*, *Stfa2*, and *Stfa2l1* presented restricted expression patterns (Figures 2B and S1). Several stefin members were expressed in the hematopoietic organs (e.g. bone marrow, spleen, thymus, and fetal liver) (Figure S1 and 2B). *Stfa2l1* expression was weak or undetectable in most tissues tested, with the notable exception of the fetal liver, bone marrow, and spleen which presented high expression levels (Figure S1 and 2B). *Stfa2l1* and *Csta* (stefins) gene expression patterns in adult mouse tissues were similar to the one observed for *Stfa3* (stefin) (Figure 2B). According to the clustering of the qRT-PCR results, *Stfa1* & *Stfa2* (stefins) gene expression profiles shared similarity with the ones observed for *Cst6* & *Cst10* (type 2 cystatins) in adult mouse tissues (Figure 2B). In selected embryonic tissues, *Stfa2l1*, *Stfa2*, and *Stfa3* expression profiles clustered together while *Csta* expression pattern shared similarity with other cystatin genes (Figure S1). Although *Stfa1* (as well as *BC117090* and *BC100530*) mapped to the same chromosomal cluster as *Stfa2l1*, *Stfa2* and *Stfa3* (Table 1) and presented high similarity at the amino acids sequence level (Figure 2A), its expression was distinctively restricted to E14.5 fetal liver cells among the tissues analyzed (Figure S1 and 2B). Together these results demonstrate that even though stefin and cystatin genes map predominantly to distinct chromosomal clusters and encode two divergent sets of proteins, they present an assortment of expression profiles sharing both intra- and inter-group similarity in mouse tissues. Among the genes covered by the deletion (*Del^16qB3Δ/+^*), *Fam162a* and *Ccdc58* are expressed ubiquitously in mouse tissues, *Csta* expression is detected in several tissues (low levels), and *Stfa2l1* is mainly expressed in hematopoietic organs.

**Table 1:** List of type 1 & 2 cystatin genes, chromosomal localization, and corresponding protein names.

Cystatin Category	Cystatin gene name	Chr	Protein name
**Type 1 (Stefins)**	*Cstb*	10qC1	Cystatin B
	*Csta* *	16qB3	Cystatin A
	*Stfa2l1* *	16qB3	Stefin A2-like 1
	*Gm5483*	16qB3	EG433016 protein
	*Gm5416*	16qB3	Stefin A1-like 1
	*2010005H15Rik*	16qB3	Hypothetical protein LOC76770
	*Stfa1*	16qB3	Stefin A1
	*BC117090*	16qB3	Stefin A1-like protein
	*BC100530*	16qB3	Stefin A-like protein
	*Stfa2*	16qB3	Stefin A2
	*Stfa3*	16qB3	Stefin A3
**Type 2 (Cystatins)**	*Cstl1*	2qG3	Cystatin-like 1 isoform 3
	*Cst11*	2qG3	Cystatin 11
	*8030411F24Rik*	2qG3	Cystatin SC
	*Cst13*	2qG3	Cystatin 13
	*9230104L09Rik*	2qG3	Cystatin E2
	*Cst12*	2qG3	Cystatin-related epididymal spermatogenic protein 3 (RESP3)
	*Cst8*	2qG3	Cystatin 8 (cystatin-related epididymal spermatogenic)
	*Cst9*	2qG3	Cystatin 9
	*Cst3*	2qG3	Cystatin C
	*Gm14129*	2qG3	Cystatin AP4
	*Gm1330*	2qG3	Cystatin AP5
	*Cst10*	2qG3	Cystatin 10 (chondrocytes)
	*Cst7*	2qG3	Cystatin F (leukocystatin)
	*Cst6*	19qA	Cystatin E/M

*Gene deleted in *Del^16qB3Δ/+^* ESCs. Chr, chromosomal localization.

### Mutant mice are phenotypically normal, fertile, and not susceptible to tumor formation

No differences could be observed between adult homozygous mutant mice and control littermate animals in terms of general appearance (including inspection of eyes, ears, fur, claws, and teeth), body weight (18.6±3.4 g for wild-type; 19.7±3.1 g for homozygous mutant; n = 3 animals monitored) and length (8.6±0.5 cm for wild-type; 8.1±0.5 cm for homozygous mutant; n = 3 animals monitored), response to stimuli (visual, aural, pain) and neuromuscular function (grip strength test). Clinical serum biochemistry and urinalysis of representative homozygous mutant and control littermate animals did not reveal significant differences for a panel of standard clinical assays (Table 2). Comparative histological analysis of fetuses (E12.5, E14.5, and E16.5) and various tissues (bone marrow, spleen, thymus, heart, kidney, muscle, brain, salivary gland, lung, stomach, pancreas, liver, intestine, colon, testis, bulbourethral gland, seminal vesicle, uterus, ovary, and mammary gland) from adult homozygous mutant mice and control littermates did not reveal any phenotypic differences (LG, data not shown). Both male and female homozygous mutant mice were fertile, producing litters of normal size that were composed of male and female animals (data not shown). To date, homozygous mutant and control littermate animals were monitored for one year without developing symptomatic diseases or tumors. In order to induce tumor formation, a cohort of wild-type (n = 13), heterozygous mutant (n = 30), and homozygous mutant (n = 15) littermate animals were submitted to whole body γ-irradiation (600 cGy) at 5 weeks of age and monitored for symptomatic tumor formation over a period of 5 months. According to the observation time, all of the mice appeared to be healthy except for a heterozygous and a homozygous mutant mouse that died 154 days post-irradiation (unknown causes, data not shown). Overall, the general health of mutant mice appeared normal in a specific-pathogen-free (SPF) animal facility.

**Table 2:** Clinical serum biochemistry and urinalysis of mutant mice and control littermates^a^.

Assays	Units	Range	M (+/+)	M (+/−)	M (−/−)	F (+/+)	F (+/−)	F (−/−)
**Serum biochemistry**								
Total Protein	g/L	36–66	46	71	47	59	59	43
Albumin	g/L	25–48	24	39	21	24	35	22
Albumin/Globulin ratio			1.1	1.2	0.8	0.7	1.5	1.0
Glucose	mmol/L	5.0–10.7	10.6	10.2	9.7	12.1	8.2	8.9
Blood urea nitrogen	mmol/L	6.4–10.4	10.9	9.0	10.3	7.2	8.6	8.2
Creatinine	µmol/L	18–71	22	n.d.	28	13	24	16
Total Bilirubin	µmol/L	2–15	11	40	8	37	22	6
Alanine transaminase	U/L	28–132	26	197	26	39	121	67
Aspartate transaminase	U/L	59–247	929	3929	722	1080	2766	936
Alkaline phosphatase	U/L	62–209	73	97	82	56	91	93
Gamma-glutamyl transferase	U/L		7	n.d.	6	15	12	6
Cholesterol	mmol/L	0.93–2.48	2.22	n.d.	3.32	2.67	3.24	2.54
Sodium	mmol/L	124–174	151	n.d.	150	146	>250	151
Potassium	mmol/L	4.6–8.0	6.7	n.d.	6.9	13.2	>14.0	6.4
Chloride	mmol/L	92–120	110	n.d.	110	107	114	120
Calcium	mmol/L	1.47–2.35	2.20	2.15	2.30	2.40	2.60	2.08
Phosphorus	mmol/L	1.97–3.26	1.79	2.37	1.83	2.02	3.18	2.59
Magnesium	mmol/L	0.33–1.60	1.04	0.33	1.04	1.11	1.44	0.97
**Urinalysis**								
Color			yellow	yellow	yellow	yellow	yellow	Yellow
Clarity			clear	clear	clear	hazy	clear	Clear
Specific Gravity			1.015	1.015	1.020	1.020	n.d.	n.d.
pH			n.d.	n.d.	6	6	n.d.	n.d.
Leukocytes			n.d.	n.d.	negative	n.d.	n.d.	n.d.
Nitrites			n.d.	n.d.	negative	n.d.	n.d.	n.d.
Proteins			1+	1+	1+	1+	negative	Negative
Glucose			normal	normal	normal	normal	normal	Normal
Ketones			negative	negative	negative	negative	n.d.	n.d.
Urobilinogen			n.d.	n.d.	normal	n.d.	n.d.	n.d.
Bilirubin			n.d.	n.d.	negative	negative	n.d.	n.d.
Blood/Hemoglobin			1+	negative	negative	1+	n.d.	n.d.

a Clinical serum biochemistry and urinalysis were performed with different sets of representative animals. M, male; F, female; +/+, wild-type; +/−, heterozygous *Del^16qB3Δ/+^*; −/−, homozygous *Del^16qB3Δ/16qB3Δ^* ; n.d., not determined.

### Functional characterization of hematopoietic lineages

**Figure 3:**
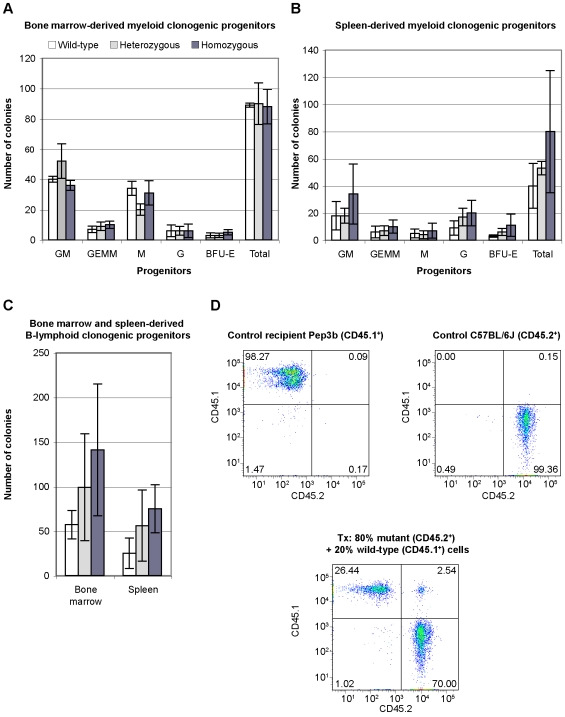
Hematopoietic system characterization. (A–C) Clonogenic hematopoietic progenitor assays. Distribution of the number of hematopoietic colonies derived from myeloid clonogenic progenitors isolated from the bone marrow (A, seeding density of 4×10^4^ cells) or the spleen (B, seeding density of 5×10^5^ cells) of n = 2 animals of each indicated genotype. GM, granulocyte-macrophage progenitor; GEMM, granulocyte-erythrocyte-macrophage-megakaryocyte progenitor; M, macrophage progenitor; G, granulocyte progenitor; BFU-E, erythroid blast-forming unit. (C) Number of hematopoietic colonies derived from B-lymphoid clonogenic progenitors isolated from the bone marrow (seeding density of 2×10^5^ cells) or spleen (seeding density of 2×10^6^ cells) of n = 2 animals of each indicated genotype. (D) Competitive hematopoietic reconstitution assay. Representative flow cytometry profiles of peripheral blood cells stained with antibodies specific to allelic forms of the cell surface marker CD45: CD45.1^+^ Pep3b recipient mouse (top left profile), CD45.2^+^ C57BL/6J control mouse (top right profile), and immunophenotyping performed 12 weeks following the transplantation (tx) of the indicated ratio of *Del^16qB3Δ/16qB3Δ^* (CD45.2^+^) and wild-type (CD45.1^+^) fetal liver cells (bottom profile). *Del^16qB3Δ/16qB3Δ^* fetal liver cells efficiently contributed to the hematopoietic reconstitution of the host (D, bottom profile, bottom right quadrant). Note that the small proportion of double positive CD45.1^+^ CD45.2^+^ cells (D, bottom profile, upper right quadrant) represents a technical artefact; hematopoietic cells in reconstituted animals are exclusively positive for one of the two CD45 allelic forms.

Since the four genes encompassed by the chromosomal deletion appeared to be transcriptionaly active in organs highly enriched with hematopoietic cells such as the fetal liver, the bone marrow, the spleen, and the thymus (Figures 1B and S1), a functional characterization of the hematological system was performed. First, similar clinical hematologic profiles were obtained using blood isolated from representative wild-type, heterozygous, and homozygous mutant littermate animals (Table 3). Then, adult mice were sacrificed to isolate bone marrow, spleen and thymus cells. The cellularity of these organs was similar for all littermate animals (data not shown). A series of antibodies specific to cell surface antigens allowed us to monitor the proportion of various hematopoietic lineages as well as stromal cells by flow cytometry (Table 4 and data not shown). Normal distributions of committed progenitors and differentiated cell lineages were observed in the bone marrow, spleen and thymus of mutant mice and control littermate animals (Table 4). Also, flow cytometry analysis revealed that the bone marrow cell compartments enriched in long-term repopulating hematopoietic stem cells, defined here as KLS (c-Kit^+^ Sca1^+^ lineages^−^) or C150^+^CD48^−^ lineages^−^ populations [25], were similar in mutant and wild-type littermate animals (Table 4). Because cathepsins regulate major histocompatibility complex II (MHC II) antigen processing and stefins are cathepsin inhibitors [26], MHC II expression was monitored on *Del^16qB3Δ/16qB3Δ^* dendritic cells (CD45^+^ CD11c^+^) using IAb immunostaining and flow cytometry. Normal cell surface expression of MHC class II molecules was observed on dendritic cells isolated from the thymus of mutant and control littermate animals (data not shown). In addition, myeloid and B-lymphoid clonogenic progenitor assays performed with cells isolated from the bone marrow and spleen of mutant and control mice did not reveal any qualitative or quantitative anomalies (Figure 3A–C). Finally, the long-term reconstitution capacity of hematopoietic stem cells isolated from E14.5 fetal livers was evaluated functionally by a competitive reconstitution assay. Homozygous mutant fetal liver cells (expressing the common leukocyte antigen CD45.2) from two mice were independently mixed at 80% ratio with wild-type fetal liver cells (CD45.1^+^) and reintroduced into 3 to 5 sublethally irradiated hosts (CD45.1^+^) together with a defined number of wild-type bone marrow helper cells (CD45.1^+^) to assist in short-term reconstitution. The hematopoietic chimerism (proportion of circulating CD45.1^+^ or CD45.2^+^ cells) in the peripheral blood was assessed by flow cytometry twelve weeks following bone marrow transplantation. When transplanted in a competitive setup (i.e., 80% mutant CD45.2^+^+20% control competitor CD45.1^+^ cells), homozygous mutant fetal liver cells contributed at high level to the hematopoietic system of the hosts (69.46±4.34% CD45.2^+^, see Figure 3D for a representative flow cytometry profile). These observations revealed that homozygous mutant hematopoietic stem cells with long-term repopulating capacity could efficiently home and function in the hematopoietic organs of the transplanted hosts. Together, these results suggest that the hematopoietic system of the homozygous *Del^16qB3Δ/16qB3Δ^* mutant mice is normal.

**Table 3 pone-0007500-t003:** Clinical hematological studies of wild-type and mutant littermate animals.

Assays	Units	M (+/+)	M (+/−)	M (−/−)	F (+/+)	F (−/−)
Hematocrit	L/L	0.464	0.471	0.489	0.461	0.482
Hemoglobin	g/L	161	163	168	163	166
Red blood cells	×10^12^/L	10.27	10.24	11.07	10.20	10.87
Mean cell volume	fL	45	46	44	45	44
Mean cell hemoglobin	pg	15.6	16.0	15.2	16.0	15.2
Mean cell hemoglobin concentration	g/L	346	347	344	353	344
White blood cells	×10^9^/L	7.2	10.7	6.1	6.9	9.8
Neutrophils	%	56	62	53	18	39
Lymphocytes	%	41	36	46	78	59
Monocytes	%	1	0	1	0	0
Eosinophils	%	2	2	0	4	2
Band	%	0	0	0	0	0
Metamyelocyte	%	0	0	0	0	0
Myelocyte	%	0	0	0	0	0
Promyelocyte	%	0	0	0	0	0
Other	%	0	0	0	0	0
Blast	%	0	0	0	0	0
Neutrophils	×10^9^/L	4.03	6.63	3.23	1.24	3.82
Lymphocytes	×10^9^/L	2.95	3.85	2.81	5.38	5.78
Monocytes	×10^9^/L	0.07	0.00	0.06	0.00	0.00
Eosinophils	×10^9^/L	0.14	0.21	0.00	0.28	0.20
Band	×10^9^/L	0.00	0.00	0.00	0.00	0.00
Metamyelocyte	×10^9^/L	0.00	0.00	0.00	0.00	0.00
Myelocyte	×10^9^/L	0.00	0.00	0.00	0.00	0.00
Promyelocyte	×10^9^/L	0.00	0.00	0.00	0.00	0.00
Other	%	0.00	0.00	0.00	0.00	0.00
Blast		0.00	0.00	0.00	0.00	0.00
Platelets	×10^9^/L	549	722	882	628	769
Poikilocyte		occ	occ	occ	occ	2+

M, male; F, female; +/+, wild-type; +/−, heterozygous *Del^16qB3Δ/+^*; −/−, homozygous *Del^16qB3Δ/16qB3Δ^* ; occ, occasional.

**Table 4:** Proportion of hematopoietic progenitors and differentiated cells in bone marrow, spleen, and thymus analysed by flow cytometry^a^.

	Wild-type	Heterozygous	Homozygous
	(%)	(%)	(%)
**Bone marrow**			
Megakaryocytic-erythroid progenitors (CD41^+^ Ter119^+^)	0.63±0.19	0.60±0.30	0.52±0.11
Megakaryocytes (CD41^+^ Ter119^−^)	5.39±0.96	4.07±0.76	4.13±0.83
Erythroblasts (CD71^+^ Ter119^+^)	23.83±3.80	27.88±0.29	25.38±5.09
Erythrocytes (CD71^−^ Ter119^+^)	1.31±1.34	0.27±0.24	0.60±0.49
Granulocyte-macrophage progenitors ( Gr1^+^ CD11b^+^)	18.42±2.28	18.11±2.05	20.30±2.38
Granulocytes (Gr1^+^ CD11b^−^)	1.44±0.12	1.26±0.47	1.42±0.17
Macrophages (Gr1^−^ CD11b^+^)	1.45±0.25	1.26±0.13	1.21±0.04
Pre-B cells (CD43^+^ B220^+^)	0.80±0.31	0.60±0.19	1.25±0.13
Mature B cells (CD43^−^ B220^+^)	34.10±3.33	26.78±4.55	26.19±8.86
T cells (CD4^+^ CD8^+^)	0.11±0.03	0.20±0.10	0.15±0.01
T cells (CD4^+^ CD8^−^)	1.09±0.15	1.37±0.51	0.76±0.09
T cells (CD4^−^ CD8^+^)	0.36±0.30	0.27±0.23	0.12±0.04
HSC- enriched KLS (c-Kit^+^ Sca1^+^ Lin^−^ b)	0.129±0.103	0.079±0.013	0.077±0.028
HSC-enriched CD150^+^ CD48^−^ Lin^−^ b	0.025±0.008	0.091±0.075	0.030±0.016
**Spleen**			
Granulocyte-macrophage progenitors and macrophages (CD11b^+^)	1.16±0.22	1.89±0.01	1.61±0.56
Pre-B cells (CD43^+^ B220^+^)	0.57±0.37	0.61±0.25	0.89±0.69
Mature B cells (CD43^−^ B220^+^)	64.21±2.45	60.14±2.53	49.17±8.74
T cells (CD4^+^ CD8^+^)	0.06±0.03	0.15±0.01	0.12±0.09
T cells (CD4^+^ CD8^−^)	16.79±1.07	15.69±1.95	20.13±3.93
T cells (CD4^−^ CD8^+^)	0.64±0.69	1.71±1.44	0.78±0.87
**Thymus**			
T cells (CD4^+^ CD8^+^)	84.10±0.42	73.95±8.98	79.40±5.94
T cells (CD4^+^ CD8^−^)	10.65±0.07	16.20±1.41	13.90±3.96
T cells (CD4^−^ CD8^+^)	1.40±0.00	2.20±0.42	1.40±0.85
Dentritic cells (CD45^+^ CD11c^+^)	0.15±0.07	0.20±0.00	0.20±0.00
Stromal cells (CD45^−^)	0.72±0.10	0.55±0.16	1.00±0.85
ETP (Lin^−^ c c-Kit^Hi^ CD25^Lo^)	0.014±0.007	0.017±0.008	0.019±0.012
DN2 T cells (Lin^−^ c c-Kit^Hi^ CD25^Hi^)	0.028±0.015	0.035±0.031	0.043±0.017
DN3 T cells (Lin^−^ c c-Kit^Lo^ CD25^Hi^)	1.35±0.20	0.94±0.56	1.32±0.01

a Cellular populations were gated to exclude debris, dead cells and enucleated erythrocytes. Two mice of each genotype were analyzed (one male and one female).

b Depletion of erythroid (Ter119), granulocytes (Gr1), and B cell (B220) lineages.

c Depletion of TCRαβ^+^, TCRγδ^+^, NK1-1^+^, CD8^+^, CD3^+^, B220^+^, CD11b^+^, CD11c^+^, Gr1^+^ and Ter119^+^ cells. ETP, early T-cell precursor; DN2 & DN3, double negative phase 2&3; Hi, high expression level; Lo, low expression level.

### Transcriptional profiling of mutant hematopoietic tissues

**Figure 4:**
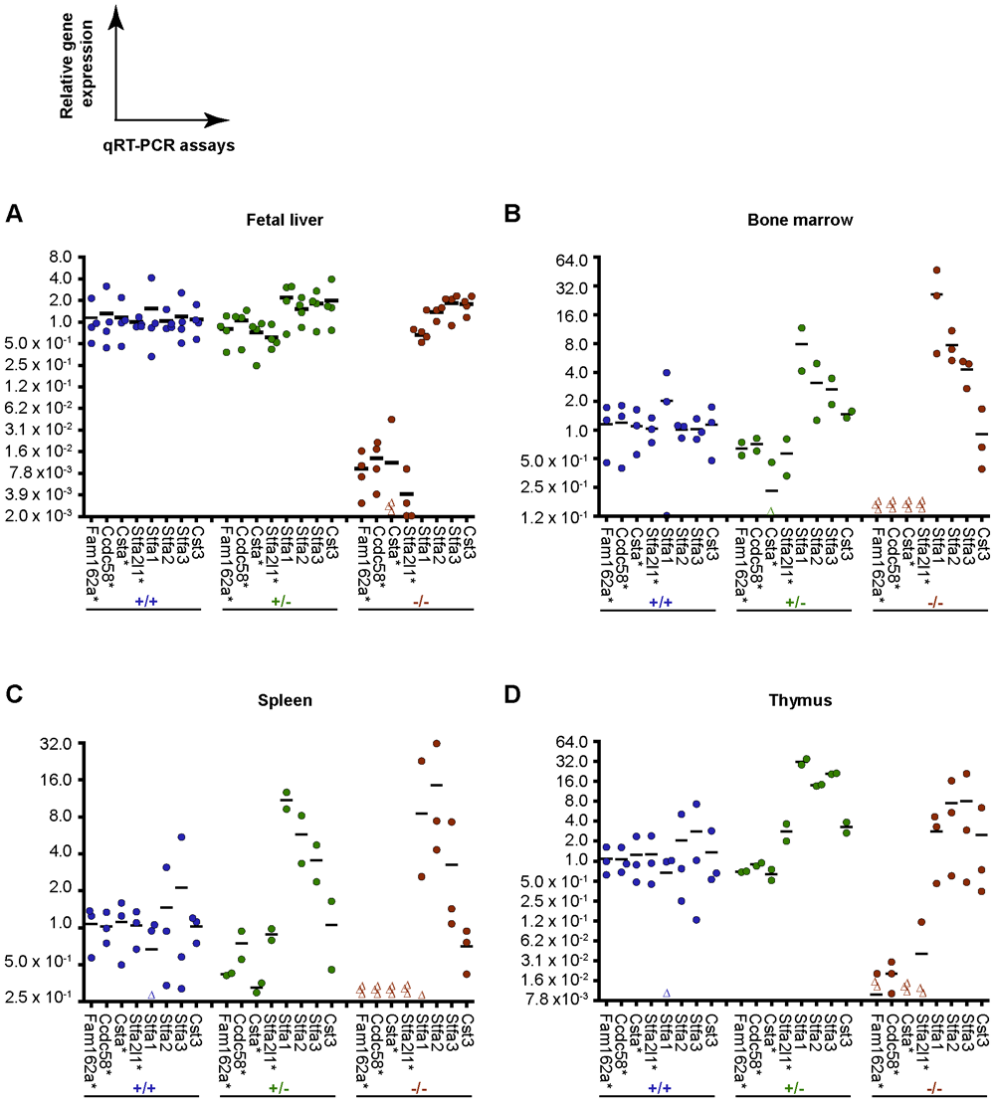
Comparative expression profiling of wild-type and mutant hematopoietic tissues. (A–D) Relative gene expression monitored by qRT-PCR using RNA extracted from hematopoietic tissues of 2–4 independent mice for each indicated genotype (+/+, wild-type; +/−, heterozygous *Del^16qB3Δ/+^*; and −/−, homozygous *Del^16qB3Δ/16qB3Δ^*). Average expression level of each indicated gene in each wild-type tissue was used as a calibrator for normalization (see Material and Methods for details). Relative gene expression observed for each independent tissue is represented by a colored dot or a Δ when the result is below the detection level of the assay; relative gene expression average is represented by an horizontal bar. Loss of expression of *Fam162a*, *Ccdc58*, *Csta*, and *Stfa2l1* was confirmed in *Del^16qB3Δ/16qB3Δ^* mouse tissues. Distinctive increases in transcriptional levels were observed for *Stfa1*, *Stfa2*, and *Stfa3* in tissues of heterozygous *Del^16qB3Δ/+^* and homozygous *Del^16qB3Δ/16qB3Δ^* adult mice compared to wild-type (B–D), but not in the fetal livers (A). Note that *Stfa1* qRT-PCR primers also detect *BC117090* and BC100530 gene expression. *, Genes deleted in *Del^16qB3Δ/+^* ESCs.

Surprisingly, the loss of four contiguous genes in the mouse genome did not compromise embryonic development or homeostasis in adult animals. As expected, qRT-PCR analysis revealed that *Ccdc58*, *Csta*, *Stfa2l1* mRNA expression levels were significantly reduced in selected tissues (fetal liver, bone marrow, spleen, and thymus) of the homozygous *Del^16qB3Δ/16qB3Δ^* animals in comparison to control wild-type animals, confirming that these genes are completely deleted from the genome of these mice (Figure 4 A–D). Notably, deleting the first coding exon of *Fam162a* drastically decreased its transcript levels in selected tissues of homozygous *Del^16qB3Δ/16qB3Δ^* animals compared to wild-type littermates (qRT-PCR primers hybridize to exons 3 and 4, Figures 1A and 4A–D). The potential subsistence of truncated Fam162a proteins was not evaluated.

One hypothesis to explain the apparent lack of phenotypic anomalies in the homozygous *Del^16qB3Δ/16qB3Δ^* mice could be that related genes are compensating for the deleted components. According to the phylogenetic and expression profiling studies described above, the stefins appeared as candidates for sharing functions with Stefin A2-like 1 and Cystatin A (Figures 2A–B and S1).

The hematopoietic tissues (fetal liver, bone marrow, spleen, and thymus) were selected to evaluate by qRT-PCR whether potential compensation for the loss of *Stfa2l1* and *Csta* in mutant animals could occur through increased mRNA levels of *Stfa2* and *Stfa3* (expressed at significant levels in these organs) as well as *Stfa1* (highly expressed in wild-type fetal liver cells) (Figures 2B, S1, and 4A–D). *Cst3* (type 2 cystatin), which maps to a chromosomal cluster distinct from the stefin genes and was ubiquitously expressed in adult and embryonic tissues, was additionally selected as a control (Table 1, Figures 2A–B, S1, and 4A–D). Interestingly, distinctive upregulation of gene expression could be observed for *Stfa1*, *Stfa2*, and *Stfa3* in the hematopoietic tissues of adult heterozygous *Del^16qB3Δ/+^* and homozygous *Del^16qB3Δ/16qB3Δ^* mice compared to wild-type animals, but this phenomenon was not observed in the fetal liver cells (Figure 4A–D). In contrast, *Cst3* levels remained relatively constant (Figure 4A–D). Taken together, these results confirmed the lack of expression of the deleted genes (*Fam162a*, *Ccdc58*, *Csta*, and *Stfa2l1*) and the increased transcriptional levels of selected stefin genes in the hematopoietic tissues of adult *Del^16qB3Δ/16qB3Δ^* animals, suggestive of compensatory mechanisms, and thus possibly explaining the lack of observed phenotypic defect.

## Discussion

A comprehensive analysis of type 1 and 2 mouse cystatin genes was undertaken, revealing that these phylogenetically distinct sub-categories of proteins are encoded by genes that share both intra- and inter-group expression profile similarities. The optimization of specific qRT-PCR assays was critical to establish the cystatin transcriptional signature in normal mouse tissues. These assays will be suitable to assist in the characterization of cancer and other diseases modeled in mice that are associated with altered expression of cystatin genes.

In this study, a homozygous mutant mouse line (*Del^16qB3Δ/16qB3Δ^*) was generated following the successful germline transmission of a chromosomal deletion previously engineered in ESCs using a retroviral-based system of Cre-*loxP* recombination (Figure 1A–D). This illustrates how the collection of mutant ESC clones published [1], now part of a larger library named DelES (e.g. **Del**etion in **E**mbryonic **S**tem Cells, manuscript in preparation), is suitable to perform *in vivo* phenotypic studies of dominant and recessive mutations in embryonic, extraembryonic, and adult tissues. As indicated by the assays employed here, mice deficient in *Fam162a*, *Ccdc58*, *Csta*, and *Stfa2l1* appeared normal, healthy, and fertile when maintained in an SPF animal facility (Table 2–4, Figures 1 and 3). These observations were unexpected given that the four deleted genes span a vast spatio-temporal spectrum of gene expression (Figures 2B and S1). Other proteins could possibly functionally compensate for these losses. Increased expression of selected stefin genes (*Stfa1*, *Stfa2*, and *Stfa3*) was observed in the hematopoietic organs of adult mutant mice suggesting that they might be participating in compensation mechanisms (Figure 4B–D). Mutant fetal liver cells presented stable *Stfa1*, *Stfa2*, and *Stfa3* transcriptional levels potentially because of the endogenous high expression of the selected genes, functional compensation by other cystatin members, or dispensability of *Csta* and *Stfa2l1* for their functions (Figures 4A and S1). Compensation mechanisms are likely to be complex and variable according to each particular tissue and discrete developmental stage, without necessarily requiring changes at the transcriptional level. Additional genetic studies, such as the chromosomal deletion of the complete stefin gene cluster mapped on mouse chromosome 16 or the cystatin gene cluster mapped on chromosome 2, would be required to potentially uncover and dissect compensation mechanisms that might be occurring in the mutant mouse model presented here. Phylogenetic and expression studies of *Fam162a* and *Ccdc58* related family members will also be necessary.

Decreased expression level of *Csta* has been observed in particular types of human cancer such as gastric carcinoma [11], esophageal squamous cell carcinoma [10], and head and neck squamous cell carcinoma [13]. However, increased expression of *Csta* has also been observed in lung tumors and carcinomas of the oropharynx [12], [14]. Moreover, overexpression of *Csta* appears to decrease metastasis potential of mammary tumors and esophageal squamous cell carcinoma in animal models [15], [16]. In this context, it is remarkable that homozygous mutant mice monitored for one year did not spontaneously develop symptomatic tumors. Given that most cancers are generated through multi-step processes, it would be of interest to analyze the effect of the chromosomal deletion in a sensitized genetic background prone to the development of the specific types of tumors mentioned above. In this study, the effect of the chromosomal deletion was monitored in mice sensitized for tumor development by γ-irradiation. Mice have been monitored for 5 months and did not develop symptomatic tumors. Among the factors affecting the latency and the spectrum of tumors induced by γ−irradiation are the irradiation dose and the genetic background of the mice (genetic background of mouse strain as well as presence of defined genetic mutations). The γ−irradiation dose used in this study (600 cGy) has been shown to be sufficient to accelerate the development of lymphomas in *Eed* hypomorphic mutant mice prior to 200 days post-irradiation, while wild-type control mice remained healthy (note that *Eed* mutant mice were backcrossed for more than 10 generations in a C57BL/6J background) [27]. It will be of interest, following a longer latency, to analyze various irradiated mouse tissues histologically, in order to look for asymptomatic tumors as described for wild-type and p21-tumor suppressor mutant mice exposed to a whole body γ−irradiation [28].

In addition, it would be interesting to challenge the immunological response of the homozygous mutant mice since stefin proteins are reported to modulate antigen presentation by regulating cathepsins [7]. Also, the effect of the chromosomal deletion could be studied in a sensitized background of autoimmune disease which is thought to involve stefin proteins [8], [9].

Overall, this study reveals that the homozygous chromosomal deletion of *Fam162a, Ccdc58*, *Csta*, *Stfa2l1* and associated intergenic regions is permissive for the development and homeostasis of adult mice. A phylogenetic study and qRT-PCR assays were developed to globally monitor expression of type 1 and 2 cystatin genes in normal mouse tissues and to gain insight into potential compensation mechanisms involved in *Csta/Stfa2l1* mutant tissues. These new tools will be of great value to characterize mouse models of human diseases associated with aberrant cystatin gene expression.

## Supporting Information

Table S1 PCR assays used for genotyping mutant embryonic stem cells or mice (engineered deletion present on chromosome 16). ^a^A duplex reaction was set up with three primers (double concentration of the common reverse primer). ^b^PCR reactions were conducted separately.
(0.02 MB XLS)

Table S2 PCR assays to monitor the loss of the genomic segment^a^. ^a^PCR primers were designed either to flank both sides of a genomic exon or one primer was flanking a given exon and a second one was hybridizing inside the exon for specificity reasons. Chr, chromosome.
(0.02 MB XLS)

Table S3 Quantitative real-time PCR assays. ^a^Primers common to transcripts: *Stfa1*, *BC117090 and BC100530*. Nucleotides identified in blue are specific to the target genes (see gene sequence alignments in Text S1). *Gene deleted in *Del^16qB3 Δ/+^* ESCs.
(0.02 MB XLS)

Text S1 Sequence alignments of Stefin-genes cluster (chromosome 16) were used to design qRT-PCR primers specific to *Stfa1*, *Stfa2*, *Stfa2l1*, and *Stfa3* (underlined in red, see primers sequences in Table S3).
(0.24 MB RTF)

Figure S1 Gene expression during mouse embryogenesis. ΔCt heatmap representing the expression profiles of *Ccdc58*, *Fam162a*, and several cystatin genes in the indicated tissues. Sample sizes are the following: n = 1 pool of embryos (E11.5, E10.5, and E9.5); n = 4 independent E14.5 fetal livers for *Fam162a*, *Ccdc58*, *Csta*, *Stfa2l1*, *Stfa1*, *Stfa2*, *Stfa3*, and *Cst3* assays; and n = 1 E14.5 fetal liver for other qRT-PCR assays. Assays were conducted in duplicate, see Material and Methods section for details. qRT-PCR assay for *Stfa1* also detects *BC117090* and *BC100530* transcripts. ΔCt above 15 represents weak gene expression or no expression; the expression cutoff varies according to each specific qRT-PCR assay (between ΔCt 15–25). *, Genes deleted in *Del^16qB3 Δ/+^* ESCs. E, embryonic day.
(0.33 MB TIF)
