# Supplementary material for: Analysis of Blood Stem Cell Activity and Cystatin Gene Expression in a Mouse Model Presenting a Chromosomal Deletion Encompassing Csta and Stfa2l1
Source: PLoS One. 2009 Oct 19;4(10):e7500. doi: 10.1371/journal.pone.0007500 (PMC2759285; doi:10.1371/journal.pone.0007500)
Supplement: Text S1 — Sequence alignments of Stefin-genes cluster (chromosome 16) were used to design qRT-PCR primers specific to Stfa1, Stfa2, Stfa2l1, and Stfa3 (underlined in red, see primers sequences in Table S3). (0.24 MB RTF) [file pone.0007500.s004.rtf]

Color code:  	unique to transcript
		Unique to Stfa3
Unique to Stfa2 + Stfa2l1
		Differentiate between Stfa2 and Stfa2l1
		Groups Stfa1, BC100530 and BC117090
		Groups Stfa1, BC100530, BC117090 and Gm5416
                                                                                                                                            
                                    *       140         *       160         *       180         *       200         *       220         *       240       
Csta                     : -----------------CCCCATTCCCTCAGATACTGTGGTGATTAGACATTTCATAGGAGGAGTTTATGGATACCTTTAAAATCCTAAGTCTCCCTGCTTGCCAGATCATTTCTTCTCA :  103
2010005H15Rik            : CCCCCACTGAAGAGGTTCAGAACTTTCTGCAGTCAGGGTCTGTCGACAGAATGAGTACGGTGTACCATTCCTAAGGACTGCCATCCAAATTCATCATACCCTGTCGGTTACTCTTCTGGA :  240
Gm5483                   : ---------------------------------------------------------------------------------------------------------GATCATTTCTTCTAA :   15
Gm5416                   : ------------------------------------------------------------------------------------------------------------------------ :    -
BC100530                 : ------------------------------------------------------------------------------------CCGAGTCTCAGTGCTTTGCAGGATCATTTCTTCTCA :   36
Stfa1                    : ---------------------------------------------------------------------------------------------------------GATCATTTCTTCTCA :   15
BC117090                 : ---------------------------------------------------------------------------------------------------------------------TCA :    3
Stfa2                    : ---------------------------------------------------------------------------------------------------------GATCATTTCTTCTCA :   15
Stfa2l1                  : -------------------------------------------------------------------------------------------------------------ATTTCTTCTCA :   11
Stfa3                    : -----------------------------------------------------------------------------------------------------------TCATTTCTTCTCA :   13
                                                                                                                                        a t  t  t a       
                                                                                                                                                          
                                    *       260         *       280         *       300         *       320         *       340         *       360       
Csta                     : GCATCCTGCCCAGCAAAGAAGCAACTTGTGATCAGAATGATACCCGGAGGCTTGACGGAGGCCAGACCTGCCACAGCAGAAGTCCAGGAGATTGCTGACCGGGTCAAAGCACAGCTCGAA :  223
2010005H15Rik            : GTGTCCTATCGAGCAAAGAAGCAACTC---ATCAGAATGATGCCTGGAGGCTTGTCACGGGCCAGATCTGCCACACCAGAAATCCAGGAGATTGCTAATAAGGTCAAGTCACTGCTTGAA :  357
Gm5483                   : GTGTCCTATCGAGCAAAGAAGCAACTC---ATCAGAATGATGCCTGGAGGCTTGTCACGGGCCAGATCTGCCACACCAGAAATCCAGGAGATTGCTGATAAGGTTAAGTCACTGCTTGAA :  132
Gm5416                   : ------------------------------------ATGATTCTCGGAGGTGTTTCAGAGGCAAGACCTGCCACACCAGAAATCCAGGAGATTGCTAACAAGGTCAGACCTCAGCTTGAA :   84
BC100530                 : GTGTCCAAGCCAGCAAAGAAGCAACTC---ATCAAGATGAGTCTCGGAGGTGTTTCAGAGGCAAGCCGTGCCACACCAGAAATCCAGAAGATTGCTGACAAGGTCAGACCTCAGCTTGAA :  153
Stfa1                    : GTGTCCAAGCCAGCAAAGAAGCAACGC---ATCAAGATGAGTCTTGGAGGTGTTTCAGAGGCAAGCCGTGCCACACCAGAAATCCAGATGATTGCTAACAAGGTCAGACCTCAGCTTGAA :  132
BC117090                 : GTGTCCAAGCCAGCAAAGAAACAAGTC---ATCAAGACTATGTACGGAGGTGTTTCAGAGGCCAAACCTGCCACACCAGAAATCCAGAAGATTGCTGACAAGGTCAGATCTCAGCTTGAA :  120
Stfa2                    : GCACCCTGCCCAGCAATGACTGAATAC---ACCATAGAAATAATTGGAGGTTTGTCAGAGGCCAGACCTGCCACATCAGAAATCCAGGAGATTGCTGACAAGGTCAGACCACTGCTTGAA :  132
Stfa2l1                  : GCACCCTGCCCAGCAATGACTGAATAC---ACCAGAAAAATAAAGGGAGGCTTGTCAGAGGCCAGACCTGCCACATCAGAAATTCAGGAGATTGCTGACAAGGTCAGACTACTGCTTGAA :  128
Stfa3                    : GCACCCTGCCCATCAATGAGTCAAGAA---AACTTAAAAATAAAGGGAGGCCTGTCAGAGGCCAGACCTGCCACACCAGAAATCCAGATGATTGCTGACAAGGTCAGACCTCTGCTTGAA :  130
                           g   cc   c agcaa ga   aa      a ca  a  At    GGAGG  T tCagaGGC AgaccTGCCACA CAGAAaTcCAG aGATTGCT AcaaGGTcA a c C GCTtGAA       
                                                                                                                                                          
                                    *       380         *       400         *       420         *       440         *       460         *       480       
Csta                     : GAGGAAACCAATGAGAAATATGAAATATTCAAAGCCGTTGAGTATAAAACTCAAGTTGTCGCTGGAGTCAATTACTTCATTAAGATGGATGTAGGGGGTGGTTGTTTCACCCACATAAAA :  343
2010005H15Rik            : GAGAAAACCAATGAGAAATATGAAGTGTTCAAAGCTGTTGAGTATAAATCTCAAGTCGTCGCTGGACAAAATTACTTCATTAAGATGGATGTTGGTGGTGGTTGTTTCCTCCACATAAAA :  477
Gm5483                   : GAGAAAACCAATGAGAAATATGAAGTGTTCAAAGCTGTTGAGTATAAATCTCAAGTCGTCGCTGGACAAAATTACTTCATTAAGATGGATGTTGGTGGTGGTTGTTTCCTCCACATAAAG :  252
Gm5416                   : GCGAGAACCAGTGAGAAATATGAAAAATTCGAAGCCGTTGAGTATAAATCTCAATTCGTCGCTGGACGAAATTATTTCATTAAGATGGATGTAGGGTGTGGTTGTTTCCTTCACATAAAA :  204
BC100530                 : GCAAAAACCAATAAGAAATATGAAAAATTCGAAGCCGTTGAGTATAAAACTCAAGCCGTCGCTGGAGAAAATATCTTCATTAAGATGGATGTAGGTCATGGTTGTTTCATTCACATAAAA :  273
Stfa1                    : GCAAAAACCAATAAGAAATATGAAAAATTCGAAGCCGTTGAGTATAAAACTCAAGTCGTCGCTGGAGAAAATATCTTCATTAAGATGGATGTAGGTCATGGTTGTTTCATTCACATAAAA :  252
BC117090                 : GCAAAAACCAATAAGAAATATGAAAAATTCGAAGCCGTTGAGTATAAAACTCAAGCCGTCGCTGGAGAAAATATCTTCATTAAGATGGATGTAGGTCATGGTTGTTTCATTCACATAAAA :  240
Stfa2                    : GAGAAAACCAATGAGAAATATGAAAAATTCAAAGCCATCGAGTATAAAGTTCAAGTCGTCCAAGGACTAAATTACTTCATTAAGATGAATGTAGGACGTGGTTGCTACCTCCACATAAAC :  252
Stfa2l1                  : GAGAAAACCAATGAGAAATATGAAAAATTCAAAGCCATCGAGTATAAAGTTCAAGTCGTCCAAGGACTAAATTACTTCATTAAGATGGATGTAGGACGTGGTTGTTACCTCCACATAAAT :  248
Stfa3                    : GAGCAAACCAATGAGAAATATGAAAAATTCGAGGCTGTCGAGTATAAATCTCAAGTCGTTGCTGGACAAAATTTGTTCATTAAGATAGATGTAGGGAATGGTTGTTTCCTTCACATGAAA :  250
                           G  aaAACCAaT AGAAATATGAAa aTTC AaGC gT GAGTATAAA cTCAAgtcGTcgctGGA  aAAT  cTTCATTAAGATggATGTaGG   TGGTTGtTtC t CACATaAA        
                                                                                                                                                          
                                    *       500         *       520         *       540         *       560         *       580         *       600       
Csta                     : GTCTTCAAGGATCTTTCTGGAAAGAATAATTTGGAACTTACTGGTTACCAGACTAACAAAACCGAGGATGATGAGCTGACCTACTTCTAAGCAGCAAATTCTAAAGTGACCTGATTCCT- :  462
2010005H15Rik            : GTCTTCACAGGGATTTCTGGAGAAAATGTCTTGGAACTTAGTGGTTACCAGACTAACAAAACCAGGAAAGATGAGCTGTCCTACTTCTAAGCAGCAAATTCTAAAGTGTCCTGACTCTT- :  596
Gm5483                   : GTCTTCAAAGGGATTTCTGGAGAAAATGTTTTAGAACTTCATGGTTACCAGACTAACAAAACCAGGAAAGACGAGCTGTCCTACTTCTAAGCAGCAAATTCTAAAGTGACCTGATT---- :  368
Gm5416                   : GTCTACCGTGCACATTCTGGAAAAGATAATTTTGAACTTCATGGTTACCAAACGAACAAAACCAAGACTGATGAGTTGACCTACTTCTAA------------------------------ :  294
BC100530                 : GTCTTCAATGGACCTACTGGAAAAGATAATTATGAACTTCATGGTTACCAGACTGACAAAACCAAGGATGATGAGCTGACCTACTTCTAAGCAGCAAATTCTAAAGTGACCTGATTCCT- :  392
Stfa1                    : GTCTTCAATGGACCTACTGGAAAAGATAATTATGAACTTCATGGTTACCAGACTGACAAAACCATGGATGAAGAGCTGACCTACTTCTAAGCAGCAAATTCTAAAGTGATCTGATTCCT- :  371
BC117090                 : GTCTTCAGTGGACCTACTGGAAAAGATAATTATGAACTTCATGGTTACCAGACTGACAAAGCCAAGGATGACGAGTTGACCTACTTCTAAGCAGCAAATTCTAAAGTGATCTGATTCCT- :  359
Stfa2                    : GTCTTAAGTGGTATTTCCAGTGAAAACGATTTGGAACTCACTGGTTATCAGACTAACAAAGCAAAGAATGATGAGCTGACCTACTTCTAAACAGCAGATTCCAACTTGACCTGATCCCCT :  372
Stfa2l1                  : GTCTTAAGTGGTATTTCCAGTGAAAACGACTTGGAACTCACTGGTTATCAGACTAACAAAGCAAAGAATGATGAGCTGACCTACTTCTAAACAACAGATTCCAACTTGACCTGATTCCCC :  368
Stfa3                    : GTCTTCAGAGGCCTTTCTGGAGAAGATGATTTGAAACTTAAGGGTTACCAGACTAACAAAACCAAGACTGATGAGCTGACCTCCATGTAAGCAGCCAATTCTAAAGTGACCTGATTCTT- :  369
                           GTCTtca  Gg   T CtgGa Aa At atT  gAACTt  tGGTTAcCAgACt ACAAA Cca G atGA GAGcTGaCCTaCtTcTAA cagca attc aa  tga ctgattc          
                                                                                                                                          


                                    *       620         *       640         *       660         *       680         *       700         *       720       
Csta                     : ----CTCATTGTAAACTGATTCGGCCATCAATAAAGAAATATTCTCCAAATAACTGTTTACTTCCCTTGTTTCTCAGTATTGTGTCAACCTTTACTTTGTAAACAATTGTTTTTAAACAC :  578
2010005H15Rik            : ----CTCCTTGTAAACAGATTTGGCCATCAATAAAGAATCAATCTTG------------------------------------------------------------------------- :  639
Gm5483                   : ----CTTCTTGTAAACAAATTTAGCCTTCAATAAAGAAATATTCTTGAAA---------------------------------------------------------------------- :  414
Gm5416                   : ------------------------------------------------------------------------------------------------------------------------ :    -
BC100530                 : ----CTCAGTGGAAAGAGATTCAGCCATGAATAAAGAAATATTCCTCAACTAAAAAAAAAAAAAA------------------------------------------------------- :  453
Stfa1                    : ----CTCAGTGGAAAGAGATTCAGCCATGAATAAAGAAATATTCCTCAACT--------------------------------------------------------------------- :  418
BC117090                 : ----CTCAGTGGAAAGAGATTCAGCCATGAAT---------------------------------------------------------------------------------------- :  387
Stfa2                    : AACCCCCATTGTAAAGAGATGCGGCCATCAATAAAGAAGCATTTTTGAAAT--------------------------------------------------------------------- :  423
Stfa2l1                  : CCCCCCCATTGTAAAGAGATGCGGCCATCAATAAAGAAACATTTTT-------------------------------------------------------------------------- :  414
Stfa3                    : ----CTCCTTGTAAAGAGATGCAGCCGTCAATAAAGAAGCATTGTTC------------------------------------------------------------------------- :  412
                               c c  tg aaa agat   gcc t aataaagaa  a t                                                                                    
