# Supplementary figures and images for: Analysis of Blood Stem Cell Activity and Cystatin Gene Expression in a Mouse Model Presenting a Chromosomal Deletion Encompassing Csta and Stfa2l1
Source: PLoS One. 2009 Oct 19;4(10):e7500. doi: 10.1371/journal.pone.0007500 (PMC2759285; doi:10.1371/journal.pone.0007500)

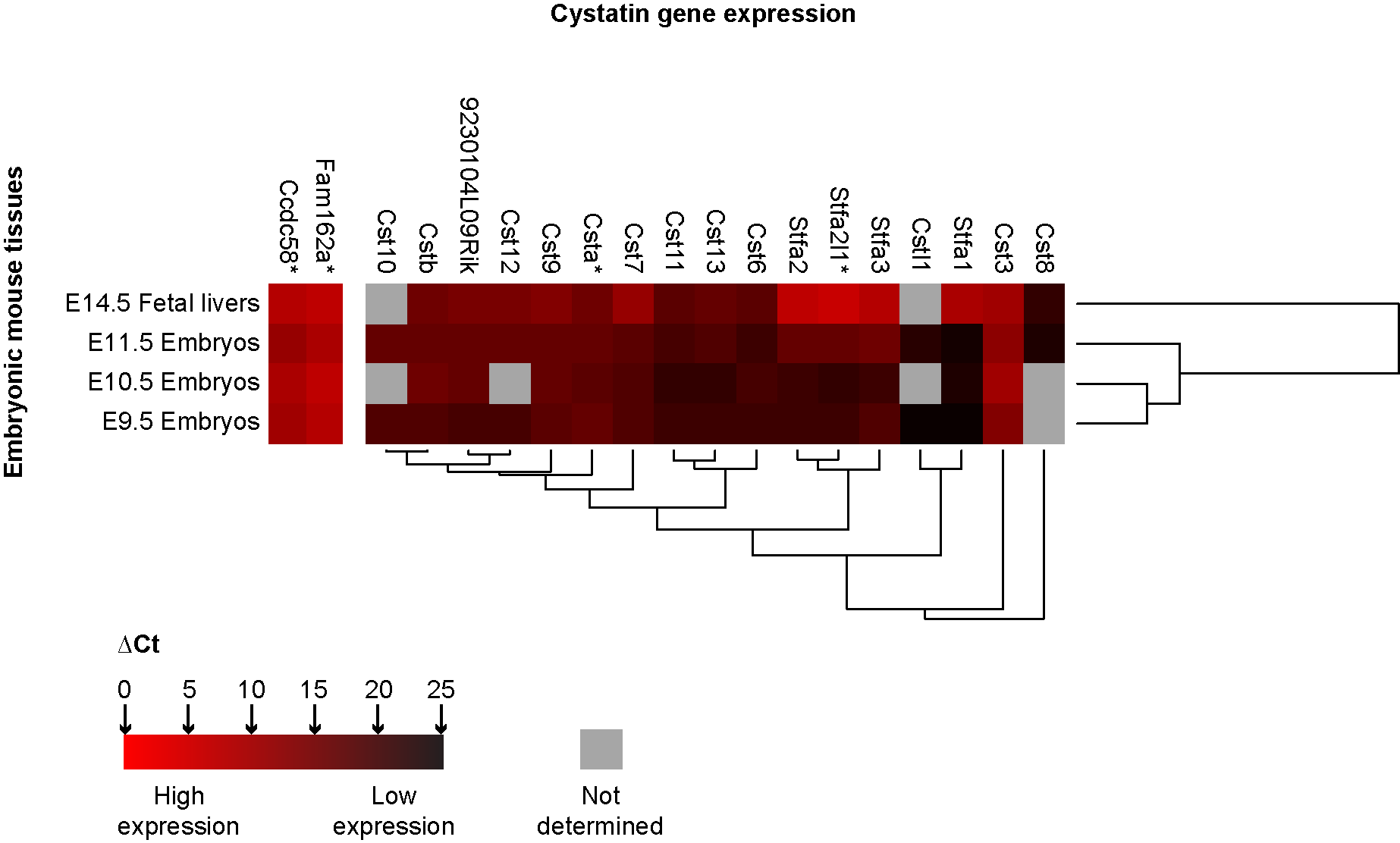

Supplement: Figure S1 — Gene expression during mouse embryogenesis. ΔCt heatmap representing the expression profiles of Ccdc58, Fam162a, and several cystatin genes in the indicated tissues. Sample sizes are the following: n = 1 pool of embryos (E11.5, E10.5, and E9.5); n = 4 independent E14.5 fetal livers for Fam162a, Ccdc58, Csta, Stfa2l1, Stfa1, Stfa2, Stfa3, and Cst3 assays; and n = 1 E14.5 fetal liver for other qRT-PCR assays. Assays were conducted in duplicate, see Material and Methods section for details. qRT-PCR assay for Stfa1 also detects BC117090 and BC100530 transcripts. ΔCt above 15 represents weak gene expression or no expression; the expression cutoff varies according to each specific qRT-PCR assay (between ΔCt 15–25). *, Genes deleted in Del16qB3 Δ/+ ESCs. E, embryonic day. (0.33 MB TIF) [file pone.0007500.s005.tif]
